# Optimising the use of SARC-F for the identification of muscle weakness by considering alternative cut-points: findings from the Newcastle SarcScreen project

**DOI:** 10.1007/s41999-023-00850-6

**Published:** 2023-08-22

**Authors:** Mo Osman, Miles D. Witham, Avan A. Sayer, Rachel Cooper

**Affiliations:** 1 AGE Research Group, Translational and Clinical Research Institute, Faculty of Medical Sciences, Newcastle University, Biomedical Research Building, Campus for Ageing and Vitality, Newcastle upon Tyne, NE4 5PL UK; 2 NIHR Newcastle Biomedical Research Centre, Newcastle upon Tyne NHS Foundation Trust, Cumbria Northumberland Tyne and Wear NHS Foundation Trust and Newcastle University, Newcastle upon Tyne, UK

**Keywords:** Sarcopenia, SARC-F, Screening, Cut-points, Obesity

## Abstract

**Purpose:**

We assessed the impact of applying different SARC-F cut-points for the identification of muscle weakness in an older clinical population.

**Methods:**

We included 159 men and 311 women aged 56–98 years who had completed the SARC-F questionnaire and had their maximum grip strength measured at an Older People’s Medicine Day Unit. We applied cut-points of ≥ 4, 3 and 2 to SARC-F and tested agreement with muscle weakness (grip strength < 27kg men, < 16kg women) in analyses stratified by sex and obesity status.

**Results:**

Prevalence of muscle weakness was 86.8% and 82.6% in men and women, respectively. Sensitivity of the SARC-F increased at lower cut-points (e.g. 81% for ≥ 4 vs 97% for ≥ 2 in women). There was typically greater sensitivity among women than men and among those classified as obese vs non-obese.

**Conclusions:**

These findings suggest that different cut-points may be required to optimise the utility of SARC-F for identifying muscle weakness in different patient sub-groups.

**Supplementary Information:**

The online version contains supplementary material available at 10.1007/s41999-023-00850-6.

## Introduction

SARC-F is a rapid, inexpensive screening tool designed to case-find sarcopenia in older adults [1]. It is a simple questionnaire aimed at identifying individuals with likely sarcopenia based on self-reports of five cardinal features and consequences of sarcopenia: low strength (difficulty lifting and carrying 10 pounds), requirement for assistance in walking, difficulty rising from a chair or bed, difficulty climbing a flight of 10 stairs, and falls. The five questions (each coded 0, 1, 2) are summed to create a total SARC-F score ranging from 0 (no difficulties) to 10 (severe difficulties). The study team who devised the SARC-F tool recommended using a cut-point of ≥ 4 to identify individuals with probable sarcopenia. These individuals were then recommended to have further assessment, involving objective measurement of muscle strength and quantity, to confirm sarcopenia.

The utility of SARC-F as a screening tool for sarcopenia has recently been questioned. A meta-analysis of 29 studies published in 2021 [2] found that while SARC-F had good reliability, it had low to moderate sensitivity (28.9–55.3%) and so may miss a significant proportion of individuals with sarcopenia. The authors concluded that SARC-F was suboptimal as a screening tool, and that assessment for sarcopenia should be undertaken without screening in high-risk groups.

The results of the above meta-analysis are based on the assessment of the recommended cut-point for SARC-F of ≥ 4, with the majority of studies testing this in relation to sarcopenia operationalised using data on both grip strength and lean mass or lean mass only. However, a recent study [3] found that when using SARC-F to identify probable sarcopenia (defined as low grip strength), application of a cut point of ≥ 1 improved sensitivity from 15 to 65% (compared with a cut-point of ≥ 4) in a general community-dwelling population. This suggests that SARC-F could still have utility in clinical practice and research for the identification of probable sarcopenia i.e. muscle weakness, but that the most appropriate cut-point to use may vary depending on the characteristics of the population. Of particular note is that variation in the utility of the SARC-F score by sex has rarely been investigated despite sex differences in the distribution of SARC-F scores and grip strength.

Another recent study [4] highlighted the importance of further work to refine diagnostic criteria and screening for sarcopenia in older adults with obesity. They concluded that the SARC-F screening tool had a low positive predictive value for sarcopenia (PPV = 37.9%) in individuals with obesity and suggested that further research was required to improve current screening and diagnostic criteria in this group.

The aim of this study was therefore to examine differences in the performance of SARC-F as a screening tool for muscle weakness in a clinical population at high-risk of sarcopenia when different cut-points are applied and to assess variation by sex and obesity status.

## Methods

### Study population

We used anonymised data from the Newcastle SarcScreen project, the key characteristics of which are described in detail elsewhere [5]. In summary, this project involved all new patients who attended the Older People’s Medicine Day Unit, a specialist service at Newcastle upon Tyne Hospitals (NuTH) NHS Foundation Trust in the North-East of England, between June 2018 and March 2020.

### Measures

As part of their visit to the Day Unit patients underwent grip strength testing. This included two measurements per hand using a Jamar hydraulic dynamometer following a standardised protocol [6] with the highest measurement used for analyses. Muscle weakness was classified as < 27 kg for men and < 16 kg for women as per European Working Group on Sarcopenia in Older People-2 criteria [7]. Patients also completed the SARC-F questionnaire, and a series of binary variables were created by applying cut-points to the total SARC-F score of ≥ 4, 3 and 2. Height and weight (either measured by nurses in clinic or self-reported) were used to calculate body mass index (BMI) (kg/m^2^). Patients were classified as obese if BMI ≥ 30 kg/m^2^ [8]. Data from SarcScreen proformas were compiled into a spreadsheet and stored within the hospital IT network with approval from the local Caldicott Guardian.

### Compliance with ethical standards

Data used in this study were collected as part of routine clinical care and made available for research with approval from the local Caldicott Guardian. Data were fully anonymised prior to analyses being undertaken. As such the project did not require evaluation by a research ethics committee and patients did not provide formal consent for the study. There was no new patient contact or additional data collection.

### Statistical analyses

We calculated sensitivity, specificity and Cohen’s kappa to assess the agreement between the three different binary categorisations of SARC-F and muscle weakness. These analyses were initially stratified by sex and then by obesity status. Patients who had complete data on grip strength, SARC-F score and BMI were included in our analytic sample. All analyses were undertaken using R version 4.1.1 (R Foundation for Statistical Computing; Vienna, Austria), run on Rstudio version 1.4.1717 (Posit; Boston, USA).

### Sensitivity analysis

To ensure that our results were not influenced by the exclusion of individuals who were unable to complete grip strength testing for health reasons (*n* = 6), we re-ran our main analyses with the inclusion of these patients allocated to the muscle weakness category.

## Results

A total of 552 patients attended the day unit with 82 patients excluded from our analytical sample due to missing data on BMI (*n* = 3), grip strength (*n* = 11), SARC-F score (*n* = 66), or a combination of factors (*n* = 2). The remaining 470 [159 men (33.8%) and 311 women (66.2%)], aged 56–98 years were included in our analytic sample. A total of 108 (67.9%) men and 240 (77.2%) women had a SARC-F score ≥ 4, and the prevalence of muscle weakness was 86.8% and 82.6% in men and women, respectively (see Table 1).

**Table 1:** Characteristics of patients from the SarcScreen project included in analyses (*n* = 470)

	*N* (%) or mean (SD) (as appropriate)	
	Men [*n* = 159 (33.8%)]	Women [*n* = 311 (66.2%)]
Age (years); mean (SD), range	79.5 (7.7), 58–98	80.2 (7.7), 56–97
Grip strength (kg)	18.5 (6.7)	10.9 (5.6)
Muscle weakness*		
No	21 (13.2%)	54 (17.4%)
Yes	138 (86.8%)	257 (82.6%)
*SARC-F scores*		
0	4 (2.5%)	3 (1%)
1	10 (6.3%)	14 (4.5%)
2	24 (15.1%)	21 (6.8%)
3	13 (8.2%)	33 (10.6%)
4	15 (9.4%)	33 (10.6%)
5	15 (9.4%)	31 (10%)
6	26 (16.4%)	48 (15.4%)
7	13 (8.2%)	44 (14.2%)
8	21 (13.2%)	45 (14.5%)
9	10 (6.3%)	25 (8%)
10	8 (5%)	14 (4.5%)
*SARC-F scores (applying cut-points of 2, 3 and 4)*		
≥ 4	108 (67.9%)	240 (77.2%)
< 4	51 (32.1%)	71 (22.8%)
≥ 3	121 (76.1%)	273 (87.8%)
< 3	38 (23.9%)	38 (12.2%)
≥ 2	145 (91.2%)	294 (94.5%)
< 2	14 (8.8%)	17 (5.5%)
BMI (kg/m^2^)	27.5 (5.1)	27.6 (6.8)
*Obese (i.e. BMI ≥ 30 kg/m*^*2*^*)*		
No	109 (68.6%)	220 (70.7%)
Yes	50 (31.4%)	91 (29.3%)

*Weakness = grip strength < 27 kg for men, < 16 kg for women

For all three cut-points of SARC-F examined, Cohen’s kappa indicated limited agreement [9] with muscle weakness; kappa < 0.2 for all cut-points (Table 2).

**Table 2:** Estimates of kappa, sensitivity, specificity, positive predictive value (PPV) and negative predictive value (NPV) comparing SARC-F with muscle weakness by SARC-F cut-point and sex

SARC-F cut point	Kappa	Sensitivity	Specificity	PPV	NPV
*Men*					
≥ 4	0.08	0.70	0.43	0.89	0.18
≥ 3	0.16	0.79	0.43	0.90	0.24
≥ 2	0.07	0.92	0.14	0.88	0.21
*Women*					
≥ 4	0.19	0.81	0.41	0.87	0.31
≥ 3	0.16	0.90	0.24	0.85	0.34
≥ 2	0.16	0.97	0.15	0.84	0.47
*Both sexes*					
≥ 4	0.15	0.77	0.41	0.87	0.25
≥ 3	0.16	0.86	0.29	0.87	0.29
≥ 2	0.13	0.95	0.15	0.85	0.35

SARC-F had greater sensitivity amongst women (0.81, 0.90 and 0.97) compared with men (0.70, 0.79 and 0.92) when using cut-points of 4, 3 and 2, respectively. In both men and women, sensitivity increased when lower SARC-F cut-points were applied, although this resulted in a lower specificity for both women (0.41, 0.24 and 0.15) and men (0.43, 0.43 and 0.14) when using cut-points of 4, 3 and 2, respectively.

When analyses were further stratified by obesity status (Table 3), there was evidence in both sexes of greater sensitivity among the group classified as obese than in the group classified as non-obese when applying a SARC-F cut point of 3 or 4 but differences by obesity status were less evident when using a SARC-F cut point of 2.

**Table 3:** Estimates of kappa, sensitivity, specificity, positive predictive value (PPV) and negative predictive value (NPV) comparing SARC-F with muscle weakness by SARC-F cut-point, sex and obesity status

SARC-F cut point	BMI < 30 kg/m^2^					BMI ≥ 30 kg/m^2^				
	Kappa	Sensitivity	Specificity	PPV	NPV	Kappa	Sensitivity	Specificity	PPV	NPV
*Men*										
≥ 4	0.06	0.67	0.43	0.89	0.16	0.12	0.74	0.43	0.89	0.21
≥ 3	0.15	0.78	0.43	0.90	0.22	0.20	0.81	0.43	0.90	0.27
≥ 2	0.00	0.93	0.07	0.87	0.13	0.20	0.91	0.29	0.89	0.33
*Women*										
≥ 4	0.24	0.78	0.53	0.89	0.32	0.07	0.89	0.17	0.81	0.27
≥ 3	0.19	0.88	0.31	0.87	0.33	0.10	0.96	0.11	0.81	0.40
≥ 2	0.18	0.97	0.17	0.86	0.50	0.10	0.96	0.11	0.81	0.40
*Both sexes*										
≥ 4	0.17	0.74	0.50	0.89	0.26	0.08	0.84	0.24	0.84	0.24
≥ 3	0.17	0.85	0.34	0.88	0.28	0.12	0.91	0.20	0.84	0.31
≥ 2	0.12	0.95	0.14	0.86	0.35	0.13	0.94	0.16	0.84	0.36

Excluding individuals who were unable to complete grip strength assessment due to health reasons did not affect our findings (see Supplementary tables S1 and S2).

## Discussion

In this patient population, there was evidence that the sensitivity and specificity of the SARC-F in identifying muscle weakness varied depending on the cut-point applied. Overall agreement was low but using a cut point of 2 or 3 resulted in higher sensitivity and lower specificity when compared with the cut-point of 4. Results varied by sex and obesity status suggesting that the SARC-F screening tool may be optimised by using alternative cut-points based on these patient characteristics.

In our study we found that the sensitivity of the SARC-F tool was greater than specificity. This is in contrast with findings of other studies which have suggested the reverse [2, 3]. It is possible that this is due to our focus on the identification of a specific component of sarcopenia, muscle weakness, rather than a definition of sarcopenia combining data on weakness and low lean mass which was used in many previous studies [2]. In addition, it could be attributed to differences between the characteristics of our study population and other study populations especially as our study focused on a clinical population attending an Older People’s Medical Day Unit in which the prevalence of muscle weakness was high. We therefore acknowledge that our findings may not be generalisable to populations with lower prevalence of muscle weakness including other clinical populations and community-dwelling samples.

To our knowledge, only one previous study has examined differences in findings by obesity status [4] and this highlighted the need for further investigation of the impact of obesity status on the utility of the SARC-F, which our paper addresses. A strength of our study is this investigation of differences in findings by both obesity status and sex, given few other studies have examined this despite evidence to suggest that these factors may impact on the utility of the SARC-F.

An additional strength of our study is the focus on an older outpatient clinical population at high risk of sarcopenia whose grip strength had been assessed following standardised protocols. However, due to this focus on a specific clinical population from a single centre in the UK, where most participants are white British, and the prevalence of muscle weakness is high, it is unknown whether our findings are applicable to other patient groups. This, along with the assessment of variation in findings by age group which we were unable to test due to limited statistical power, requires further investigation, ideally in a larger sample with greater statistical power. Once the generalisability of these findings has been established the next step will be to develop recommendations on the SARC-F cut-points that should be used to optimise identification of muscle weakness in different patient sub-groups.

Another limitation is that the study uses grip strength as a surrogate for sarcopenia diagnosis. As we did not have measures of muscle quantity or quality, we are unable to comment on confirmed diagnosis of sarcopenia.

## Conclusions

In a clinical population at high risk of sarcopenia there may be scope to optimise the utility of the SARC-F tool for the identification of muscle weakness by considering the use of alternative cut-points for different patient sub-groups.

Improving the sensitivity of SARC-F as a screening tool would be clinically valuable as it would ensure that more patients are tested for sarcopenia and less diagnoses are missed. This also has relevance for pre-screening and case finding for research studies where there are acknowledged challenges in identifying study participants with sarcopenia [10].

### Electronic supplementary material

Below is the link to the electronic supplementary material.

Supplementary file1 (DOCX 15 KB)
