# Supplementary material for: Optimising the use of SARC-F for the identification of muscle weakness by considering alternative cut-points: findings from the Newcastle SarcScreen project
Source: Eur Geriatr Med. 2023 Aug 22;14(6):1327–31. doi: 10.1007/s41999-023-00850-6 (PMC10754758; doi:10.1007/s41999-023-00850-6)
Supplement: Supplementary file 1 — Supplementary file1 (DOCX 15 KB) [file 41999_2023_850_MOESM1_ESM.docx]

**Optimising the use of SARC-F for the identification of muscle weakness by considering alternative cut-points: findings from the Newcastle SarcScreen project**

Mo Osman, Miles D Witham, Avan A Sayer, Rachel Cooper

**Supplementary tables**

Table S1: Sensitivity analyses - Estimates of kappa, sensitivity and specificity comparing SARC-F with muscle weakness by SARC-F cut-point and sex after including individuals that could not do grip strength assessment for health reasons (n=6)

|  | Men | | | Women | | |
| --- | --- | --- | --- | --- | --- | --- |
| SARC-F Cut Point | Kappa | Sensitivity | Specificity | Kappa | Sensitivity | Specificity |
| ≥ 4 | 0.08 | 0.70 | 0.43 | 0.20 | 0.81 | 0.41 |
| ≥ 3 | 0.16 | 0.79 | 0.43 | 0.16 | 0.90 | 0.24 |
| ≥ 2 | 0.07 | 0.92 | 0.14 | 0.16 | 0.97 | 0.15 |

Table S2: Sensitivity analyses - Estimates of kappa, sensitivity and specificity comparing SARC-F with muscle weakness by SARC-F cut-point, sex and obesity status after including individuals that could not do grip strength assessment for health reasons (n=6)

|  | BMI < 30 | | | BMI ≥ 30 | | |
| --- | --- | --- | --- | --- | --- | --- |
| SARC-F Cut Point | Kappa | Sensitivity | Specificity | Kappa | Sensitivity | Specificity |
| **Men** |  |  |  |  |  |  |
| ≥ 4 | 0.06 | 0.68 | 0.43 | 0.12 | 0.74 | 0.43 |
| ≥ 3 | 0.15 | 0.78 | 0.43 | 0.20 | 0.81 | 0.43 |
| ≥ 2 | 0.00 | 0.93 | 0.07 | 0.20 | 0.91 | 0.29 |
| **Women** |  |  |  |  |  |  |
| ≥ 4 | 0.24 | 0.78 | 0.53 | 0.07 | 0.89 | 0.17 |
| ≥ 3 | 0.20 | 0.88 | 0.31 | 0.10 | 0.96 | 0.11 |
| ≥ 2 | 0.18 | 0.97 | 0.17 | 0.10 | 0.96 | 0.11 |
